# Addressing Challenges of Distance Learning in the Pandemic with Edge Intelligence Enabled Multicast and Caching Solution

**DOI:** 10.3390/s22031092

**Published:** 2022-01-31

**Authors:** Kashif Bilal, Junaid Shuja, Aiman Erbad, Waleed Alasmary, Eisa Alanazi, Abdullah Alourani

**Affiliations:** 1 Department of Computer Science, Abbottabad Campus, COMSATS University Islamabad, Abbottabad 22060, Pakistan; kashifbilal@cuiatd.edu.pk; 2 College of Science and Engineering, Hamad Bin Khalifa University, Doha 5825, Qatar; aerbad@hbku.edu.qa; 3 Computer Engineering Department, College of Computer and Information Systems, Umm Al-Qura University, Makkah 21955, Saudi Arabia; wsasmary@uqu.edu.sa; 4 Department of Computer Science, Umm Al-Qura University, Makkah 21955, Saudi Arabia; eaanazi@uqu.edu.sa; 5 Department of Computer Science and Information, College of Science in Zulfi, Majmaah University, Al-Majmaah 11952, Saudi Arabia; a.alourani@mu.edu.sa

**Keywords:** edge intelligence, video multicast, distance learning, eMBMS, edge caching

## Abstract

The COVID-19 pandemic has affected the world socially and economically changing behaviors towards medical facilities, public gatherings, workplaces, and education. Educational institutes have been shutdown sporadically across the globe forcing teachers and students to adopt distance learning techniques. Due to the closure of educational institutes, work and learn from home methods have burdened the network resources and considerably decreased a viewer’s Quality of Experience (QoE). The situation calls for innovative techniques to handle the surging load of video traffic on cellular networks. In the scenario of distance learning, there is ample opportunity to realize multi-cast delivery instead of a conventional unicast. However, the existing 5G architecture does not support service-less multi-cast. In this article, we advance the case of Virtual Network Function (VNF) based service-less architecture for video multicast. Multicasting a video session for distance learning significantly lowers the burden on core and Radio Access Networks (RAN) as demonstrated by evaluation over a real-world dataset. We debate the role of Edge Intelligence (EI) for enabling multicast and edge caching for distance learning to complement the performance of the proposed VNF architecture. EI offers the determination of users that are part of a multicast session based on location, session, and cell information. Moreover, user preferences and network’s contextual information can differentiate between live and cached access patterns optimizing edge caching decisions. While exploring the opportunities of EI-enabled distance learning, we demonstrate a significant reduction in network operator resource utilization and an increase in user QoE for VNF based multicast transmission.

## 1. Introduction

The World Health Organization (WHO) declared the COVID-19 as a pandemic on 11 March 2020. The pandemic has spread across all countries and populated regions of the world, causing significant disruption in social, medical, and economic affairs. Social distancing and lock-down policies are adopted worldwide to stop the disease spread. The scientific community has worked tirelessly to come up with solutions for the mitigation, diagnosis, and prognosis of the pandemic. The computer science research groups have largely focused on image-based pandemic diagnosis, case report-based forecasting, and social media-based sentiment analysis. Interestingly, Machine Learning (ML) techniques are enablers of all the aforementioned research directions on COVID-19 [1].

The contiguous nature of the pandemic has forced the shutdown of workplaces, public gatherings, and educational institutes disrupting normal social behaviors. Due to the quick spread of new variants and the drastic increase in the number of cases in the second wave, lock-down, and social distancing policies have been implemented while educational institutes have resorted to distance learning all over the world. Network services have experienced a major surge in traffic due to many consequences of the pandemic including work from home, distance learning, telehealth, home-based entertainment, and sports live-streaming events. Modern 4G/5G networks have to support the increasing traffic demands with limited infrastructure upgrades. Moreover, students, in particular, may reside in areas with limited network access or congested dormitories disrupting the conduct of distance learning [2]. Innovative techniques are called for to improve resource utilization of cellular networks in the current pandemic [3].

The major part of the Internet traffic (60–80%) comprises video content. Cisco predicted that, by the year 2021, the mobile video traffic will be seven times higher than today, and the live video traffic will be at least 13 percent of the total Internet traffic [4,5]. The percentage of live video traffic is predicted to increase in the current scenario of distance learning adopted by teaching institutions around the globe. The statistics of university campuses with distance learning platforms show a huge variation in different Internet traffic patterns [6,7]. In particular, the number of MS team video calls has jumped from tens daily before the lockdown to hundreds daily after the lockdown. The studies observed that the incoming traffic to campus LAN decreased significantly while outgoing traffic increased 2.8 times due to in-campus hosting of online education platforms. With no immediate medical cure in sight, the global community is adopting online tools for education, health, business meeting, and conferences. To fairly address the bandwidth and Quality of Experience (QoE) requirements of live video streams, the emerging 5G networks need novel architectural designs [8,9].

Live video data-sets show that groups of users request the same content while residing in close vicinity [10]. Distance learning in the pandemic is an example of this situation. Each user receives a separate unicast video stream of the same content towards the RAN resulting in resource wastage. The QoE of the video streams is reduced due to overloaded network resources in the 5G RAN. The multicast delivery can reduce network resource wastage and increase user QoE. However, the identification of unicast streams that are part of live video traffic and their convergence to multicast is necessary. The convergence of edge computing with AI termed as Edge Intelligence (EI) advocates the application of intelligent caching and routing in 5G networks. The integration of AI in edge computing comes with the increasing hardware capabilities, the sophisticated wireless network properties, and the capability of AI to optimize objectives in complex network environments [11,12]. ML techniques in general, and EI specifically, are called for the identification of multicast video patterns in a service-less 5G multicast architecture [13,14].

The evolved Multimedia Broadcast Multicast Service (eMBMS) in LTE-A is a service-oriented architecture requiring pre-announcement of the video to enable multicasts. However, the live video streaming and distance learning applications can not follow a service-oriented architecture as, for each stream, the session has to be announced on each Mobile Network Operator (MNO) core. On the contrary, a service-less architecture to enhance the capabilities of live video streaming with a Virtual Network Function (VNF)-based solution has been proposed [4]. A VNF Application Server (VNF-AS) can be instantiated in an MNO core to act as a proxy for end-user and content provider (CP) and instantiate multicast service spontaneously. VNF-AS extends the capabilities of eMBMS to provide service-less multicast and reduce redundant video streams over RAN and core network. We propose VNF-AS to be complemented with the EI and capabilities of mobile edge caching to further alleviate the burden on the 5G cellular network in the context of distance learning. A group of users in the distance learning session are expected to watch recorded streams as they may be absent due to network outages. Moreover, users can experience high SNR leading to low QoE. Caching at the edge of the network can reduce redundant file access from CDNs and cloud data centers for such users. However, the user access patterns, mobility, and network conditions at the network edge need to be predicted [15,16]. Moreover, ML techniques can also provide intelligent input to the VNF-AS to optimize the decision of what, when, and where to multicast video streams based on user access patterns and content features [13,17]. Caching is essential for users of distance learning with limited network connectivity. EI can enhance the performance of edge caching with user statistics and content features as input [13,18].

The main objective of this article is to identify the opportunities of multicast and caching in the existing 5G architecture while evaluating the performance of VNF-AS for multicast distance learning. The contributions of this article are as follows:

- We propose a service-less multicast architecture enabled by VNF-AS for distance learning. We investigate the proposed VNF-AS for distance learning on a real-time dataset for traffic, QoE, and resource optimization.
- We propose and debate the opportunity of EI to enhance the performance of VNF-AS. EI can enable optimized decisions of what to cache and multicast (popular live streams), when to cache and multicast (timing of live stream and network off-peak hours), and where to cache and multicast (BSs with higher user associations).
- We propose intelligent edge caching and device-to-device (D2D) propagation to facilitate users with limited network connectivity or offline viewing behaviour while further alleviating the burden on RAN and reducing duplicate content transmissions.

The rest of the article is organized as follows: Section 2 details the related work on EI and eMBMS in 5G. In Section 3, we present the Service-less Multicast Architecture enabled by VNF-AS for COVID-19 distance learning. The proposed EI and edge caching solutions are also discussed in this section. Section 4 details the evaluation of proposed work on the real-time dataset. We debate the research challenges and future directions in Section 5. Section 6 concludes the article.

## 2. Related Work

**Figure 1:**
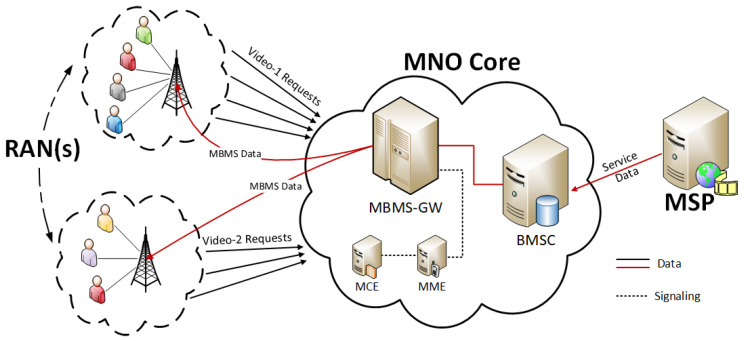
eMBMS architecture.

A wide range of services is promised to be introduced in the upcoming 5G cellular technologies. However, the 3rd Generation Partnership Project (3GPP) did not acknowledge the gravity of multicast capabilities in the 5G services as per 3GPP Release-15 (2018) termed as 5G Phase 1 specification. As the 3GPP Release-16 (2020), i.e., 5G Phase 2 specification was introduced, it was heeded that the introduction of the 5G cellular systems will provide a unified transmission platform that could deliver the unicast and broadcast/multicast services simultaneously. This can be accomplished by evolving the Multimedia Broadcast Multicast Services (MBMS) into a more proficient Point-To-Multipoint (PTM) delivery system named as 5G-MBMS or evolved MBMS (eMBMS). The eMBMS could present higher spectrum utilization using a converged system allowing dynamic spectrum allocation among different services. An overview of the eMBMS architecture is illustrated in Figure 1 [19,20].

Zhang et al. [21] propose a Non-Orthogonal Multiplexing (NOM) scheme to deliver various types of broadcast, multicast, and unicast services over an eMBMS network architecture. The theoretical analysis in accordance with the extensive computer simulations shows significant capacity gains. In particular, when a two-layer Power-based NOM (P-NOM) is exploited, then a broadcast service can be delivered on top of a unicast network without any capacity reduction. A key benefit of the used scheme, in contrast to the Non-Orthogonal Multiple Access (NOMA), is that P-NOM requires no to very little changes in the existing radio resource allocation mechanisms. In [22], researchers propose changes in the 5G core network architecture to assimilate broadcast/multicast capabilities. This article presents two architectural solutions. First, the transparent multicast transport creates a network pipe over the 5G network to support the broadcast/multicast traffic. Second, a service-based method that sets up a broadcast/multicast service to deliver the multimedia content. Both schemes need architectural changes in the 5G cellular network architecture.

In literature, most of the solutions need architectural changes to propose broadcast/multicast schemes. Researchers [23] present a D2D-based multicast communication scheme to share the available spectrum and exploit the mobile user’s resources for caching purposes without proposing any architectural changes in the 5G network. This scheme offloads the video content from cellular network to dense D2D 5G networks considering the physical and social attributes of the network users. The main goal of the proposed technique is to maximize the system capacity as well as maintaining fair Quality of Service (QoS) among the users. Another study that requires no architectural changes in the existing network architecture proposed a Virtual Network Function (VNF)-based scheme to enable multicast transmission over a unicast network [4]. This technique proposes a VNF Application Server within the core network of 5G architecture to initiate and monitor an on-the-fly multicast service. The scheme enables a video content from a Crowdsourced Live Video Provider (CLVP), i.e., a non-Multicast Service Provider (MSP), such as Facebook and YouTube to be transmitted to multiple cellular network users on a unicast network. The study shows cost and bandwidth savings as well as an increase in the QoS through extensive simulations.

Beyond multicast service on 5G networks, we focus on EI to optimize decisions of caching and multicasts. Edge networks provide proximate services to a group of users in the close geographic vicinity. Caching content within edge networks can lower access latency and reduce the number of redundant flows from the core network. Caching at the edge of the network can help 5G networks meet the ultra-low latency requirements of modern applications. Moreover, ML techniques are able to learn from their environment without the need for explicit programming. ML techniques can help edge caching while predicting user requests based on user preferences, mobility, and content popularity [12,13,24]. The convergence of AI/ML techniques with edge networks to optimize complex caching and routing decisions leads to the emergence of Edge Intelligence (EI).

Authors [15] presented a supervised ML based content edge caching technique to lower backhaul traffic in cellular networks. The authors employ ML for popularity prediction of videos where content features are taken as input. The popularity of new videos is predicted based on the similarity and popularity of old videos. A 3D convolutional neural network (CNN) with a multiple stage pooling algorithm is employed to extract spatio-temporal features of videos. The extracted features are vectorized and clustered based on cosine distances. A Support Vector Machine (SVM) algorithm classifies each video into a video category. Afterward, a supervised ML algorithm is trained to predict popularity of a video category. To the best of our knowledge, the application of EI to predict the collective opportunities of 5G multicast and edge caching has not been explored. Regression techniques were utilized in [25] to anticipate the demand of online content. The nonlinear attributes of Support Vector Regression and Gaussian Radial Basis Functions are engaged to forecast popularity patterns. Visual features, such as color, face, and scene dynamics, are selected from the videos while utilizing a DNN (ResNet-152). Temporal and social features, such as views and likes, are also utilized in the regression model. Multivariate Radial Basis Function utilizes the linear regression model for temporal popularity growth of video and nonlinear regression model to learn similarity between a set of videos. A data set of 24k videos with a daily count of temporal features over a span of one month from social platforms is used for evaluation. The regression models lead to a prediction accuracy of more than 95%. Although this work employs ML techniques, it predicts the popularity of videos from a global perspective instead of an edge network perspective.

Table 1 lists the related work and highlights their contributions. The table shows that the existing solutions are one-dimensional, enabling either multicast delivery or edge caching in 5G networks.

In this article, we present a three-dimensional solution to alleviate the burden from networks in the existing pandemic situation. Firstly, we propose a Virtual Network Function (VNF) based service-less architecture at the MNO core to enable video multicast for distance learning. We demonstrate its efficiency in terms of bandwidth consumption, resource block utilization, and QoE optimization on a real-world edge network dataset. Secondly, we propose to complement the VNF with EI to predict and learn the multicast data streams in the MNO core. Lastly, EI is conceptualized to resolve the issue of end-users with high SNR who can not watch the stream live by predicting edge caching opportunities.

## 3. A Service-Less Multicast Architecture for Distance Learning

We first describe the VNF multicast architecture in this section that forms the basis of existing proposal. Afterward, we detail the proposed EI enabled multicast and MEC caching architecture with a design and algorithm.

### 3.1. VNF Multicast Architecture

The eMBMS architecture for multicast in 5G is service oriented, resulting in unicast transmission of live video streams. Virtualization in the cellular networks brings forward an opportunity for application of multiple management functions. A VNF can perform functions such as firewalls and IPS. A VNF overlay module at 5G core is proposed to enable on-the-fly service-less multicast initiation [26]. A VNF-Application Server (VNF-AS) works as a proxy entity for both content provider and user device while residing at the edge of the network. The VNF-AS takes as input service ID, multicast IP address, and session time among other parameters provided by the UE and the multicast service provider from within an edge network. The VNF-AS monitors requests for live videos from the perspective of an edge network. If multiple requests are generated from the same cell within the edge network, VNF-AS enables multicast on-the-fly. The VNF-AS forwards these parameters to the eMBMS architecture to initiate multicast service. The UE and service provider are not inherently multicast and the VNF-AS acts as a proxy to enable multicast in 5G eMBMS architecture. Request monitoring and multicast session initiation, content provider proxy, and user equipment proxy constitute the basic modules of the VNF-AS. The VNF module sends less content requests due to its multicast nature while lowering the traffic burden from the network.

### 3.2. EI Enabled Multicast Architecture

**Figure 2:**
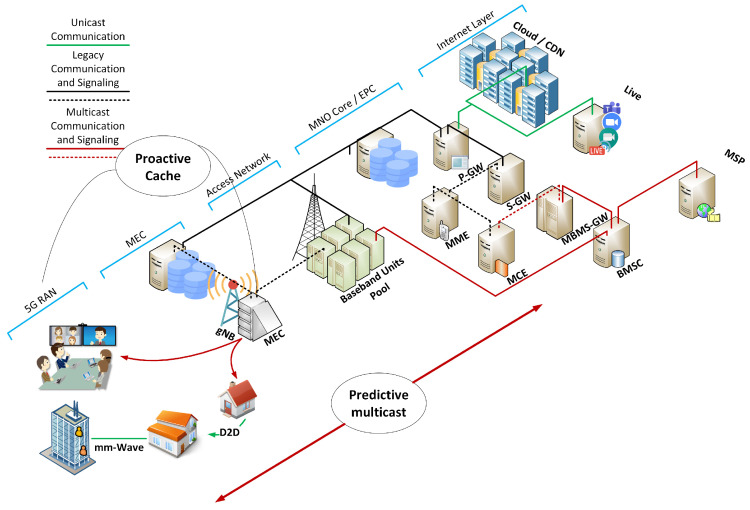
The concept of multi-cast, machine learning, and distance learning.

The pandemic has forced the world to lock down and disrupted normal life patterns including education. However, video call applications such as Zoom and MS teams accessible via wired and wireless technologies including cellular networks have facilitated online education. Most of the students taking classes at various levels, specifically at the school and college level, live within close vicinity. For instance, most of the school students live in the same city or neighborhood as the school. Moreover, most of the classes in different schools are scheduled at the same time interval, placing a considerable burden on the RAN, backhaul, and transit links for video transmissions. As the mode of communication is unicast in existing 5G architecture, many fold redundant resources are consumed at each network layer. Multicast is a feasible and inherent solution for such use cases, where most of the students are expected to watch the same stream for a long time. The existing VNF based multicast initiation module can be utilized for optimization of distance learning traffic. However, the application of EI is imperative to answer: **(a)** which unicast sessions can be merged in a multicast session based on parameters from UE and MNO, and **(b)** where edge caching of distance learning session can help users with low QoE to view content at a latter time. VNF based Multicast can be aided with machine learning and edge caching techniques for a proactive approach towards MNO resource optimization and user QoE enhancement. An overview of the stated concept is illustrated in Figure 2. The details of our system design are presented below.

- **Cold start:** The main objective of cold start is to introduce multicast without the service announcement from the service provider. We utilize the service-less multicast architecture to enable cold start multicast for distance learning. In the basic service-less multicast architecture, the online sessions are started with unicast. As users keep on joining the session, the unicast delivery is converted to multicast enabled by the VNF module. However, the cold start has several disadvantages such as: **(a)** It takes a little time to convert from unicast to multicast and **(b)** while transmitting unicast, many resources are wasted until conversion and convergence. Moreover, we collect data regarding user behavior for proactive and predictive multicast and caching in the cold start stage.
- **Predictive multicast:** In the second stage, EI is proposed to identify opportunities to proactively create a multicast session beforehand as soon as the first request arrives at the server. Proactive multicast is enabled by applying ML to group users based on their geographic locations and session times among other attributes. Unsupervised learning (clustering) techniques will be used on the collected data to group users in the same session without obtaining information from the video streaming application. The MNO may face penalties due to the unnecessary initiation of the multicast service and usage of multicast resources in case only a single request arrives.
- **Proactive Caching:** In the third stage of our proposed design, proactive caching will be used to store the recorded streams near users. The distance learning applications such as MS Teams provides access to recorded streams that can be viewed later. The user behavior may vary towards the attendance of live session based on his interest and channel conditions. Our proposed EI will learn user access patterns from their preferences to optimize edge caching. Edge caching will minimize backhaul and RAN resource usage. To achieve this, we propose user end (UE) caching which will be helpful in three scenarios: **(a)** if a cluster member is not attending the multicast stream in live mode, the stream data will be cached locally at UE if the user is connected, **(b)** in case the cluster member is not connected to the cellular network, community detection (a user from the same cluster) will be used to select a member lying in close vicinity. In case the user requests the stream later lying within the selected cache, then D2D mechanisms will be used to deliver the content, and **(c)** in case the cluster member is not connected to the cellular network, and is a geo-outlier with limited connectivity, a D2D relay network will be considered to deliver the stream.

We assume that the traffic is uni-directional due to the multicast nature. This means that, in the case of distance learning, most of the streaming traffic is directed from the source (instructor) to the destinations (students). The video can be cached at the edge as long as the cache is not full. The content removal policy that we consider is the Least Recently Used (LRU) cache replacement policy [27]. The access date of the video is updated as soon as the video is accessed by any user. Therefore, when the available storage is consumed, then the video with the least recent access date will be removed. In this way, the removed video will be one that no user has accessed for long, which depicts that this video is no longer a popular or required video. The Algorithm for the proposed solution is presented in Algorithm 1.
**Algorithm 1** Pseudo code for EI enabled multicast and caching.
    **COLD START**
    Monitor: User ID, session ID, eNB ID
    Create: UE and content provider proxy for service-less multicast in eMBMS
    Initiate: VNF based Multicast
    **PREDICTIVE MULTICAST**
    Input: Timestamps, GPS, eNB IDs and SNR
    Apply: EI to cluster users in same eNB
    Cluster: Input multi-dimensional data (GPS, eNB, timestamp) to clustering algorithms
    Output: Geo-spatial clusters with users of same session near the same eNB in same cluster
    Initiate: Proactive multicast if number of users > than a threshold
    **PROACTIVE CACHING**
    Input: User access pattern for session, SNR, User Clusters
    **if** UE not connected during Live session and not GPS outlier **then**
     Apply: Geo-spatial clustering to identify community members
     Select: A community member near to UE for caching
     Send: Share stream through D2D communication
    **end if**
    **if** UE not connected during Live session and GPS outliers **then**
     From: A D2D relay between community members
     Send: Share stream through D2D relay
    **end if**


The detailed algorithm of a cold start step to create UE and content provider proxies can be found in our previous work [26] and presented as follows. Evolved Multimedia Broadcast Multicast Services (eMBMS) is the standard service for multicast in 4G and 5G networks. eMBMS is based on a services-oriented delivery architecture, which means, if a service needs to be multicast, then the service will be announced prior. However, in crowdsourced live video streaming, the mobile network operator cannot announce a service in real time. Ref. [26] discusses the solution to this problem with a technique termed as EdgeCast. The EdgeCast is comprised of three major modules: (a) Request monitoring and multicast/unicast session initiation module, (b) content provider proxy module (CP-Proxy), and (c) user equipment proxy module (UE-Proxy). As multiple viewers watch a live video stream from the content provider at the same time, the stream monitor module analyzes every video request routing through the core network. On arrival of the request, the monitor module looks for an entry of the requested video in its tables. If found, the number of total viewers for the corresponding video is incremented by 1; otherwise, a new record is inserted and the video is streamed in a unicast fashion. Next, the requested video is searched in another table that contains the information of the videos being streamed to all eNB cells. If the entry is found in any of the records, the number of viewers of that video in that specific cell is incremented by 1, a new service ID is generated, the service ID, video tag, eNB cell ID, and lastly the multicast address is generated and the status of the requested video is set to multicast in the corresponding tables. If the eNB cell for the requested video is not found in the tables, then a new entry is created and the status of the requested video is set to unicast. The process is repeated in reverse order when a viewer leaves a video session and the feasibility of the multicast session is checked.

The algorithm for geo-temporal clustering based community detection is provided in [28]. The geo-spatial clustering algorithm is a two-step framework that performs geographic clustering based on the GPS longitude and latitude points in the first step. The geographic clusters represent the edge clusters. In the second step, temporal (session timestamps) and textual data (network operator, cell ID) are used to form sub-clusters within the geographic clusters. The sub-clusters represent the users that can receive multicast traffic for the same distance learning session.

Data from the UE and MNO are necessary for the implementation of the proposed EI framework. Several applications such as G-NetTrack can be utilized for the collection of network information [29,30]. G-NetTrack collects channel-related, context-related, cell-related, and throughput related parameters. The pro version of G-NetTrack enables collection of timestamp, GPS coordinates, velocity of mobile device, operator name, cell ID, SNR, RSSI, and CQI among other parameters. The smartphone application of G-NetTrack pro can be installed on the student devices to monitor the aforementioned parameters during distance learning activities. Currently, we are in the process of collecting a large dataset from students based on distance learning classes. As a result, the proposed framework will not require any class related information from the university and multicast sessions will be created based only on UE and network level information. The evaluation in the forthcoming section evaluates the basic proposal of VNF based multicast to shed light on bandwidth and resource optimizations from the proposed work.

## 4. Evaluation and Results

We describe the dataset utilized in the experiments and the results in subsections below.

### 4.1. Dataset

We used a publicly available real-world EUA dataset for the evaluation of the proposed VNF-AS model [31,32]. The dataset shows the exact locations of the eNBs/MECs and the cellular users of different MNOs across Australia. We chose the Melbourne Central Business District (CBD) as the Region of Interest (ROI) for our experimentation as it depicts a dense 5G cellular scenario. Using the precise locations taken from the dataset, we calculated cellular traffic load and resource usage at various levels.

From the ROI, i.e., Melbourne CBD, we selected six distant eNBs covering an area of 2.05 square kilometers depicting a dense cellular deployment. Each UE was attached within the ROI with one of the six eNBs depending on the cell radius of the eNBs. Each UE was assigned a CQI relative to its distance from the attached eNB. We streamed a 720p video of 1 h in length in time to all of the six eNBs with requesting users in both unicast and multicast modes during simulations. For the calculation of the traffic load, bandwidth, RB usage, and QoE score, we took a scenario of streaming a lecture video to various students within the ROI. We selected a random number of users from all the attached users within each cell not to lose the concept of generality. The peak hours in a cellular network has changed dramatically due to the novel COVID-19 pandemic situation. Therefore, the burden on each layer of the cellular networks has undoubtedly increased significantly. Consequently, the MNO cannot provide a higher number of resources to each user on a dedicated channel, i.e., unicast mode. In such situations where several students attend an online lecture within the radius of one cell, multicast transmission instantiated by VNF-AS can be exploited to manage the MNO resources efficiently, reducing the cost of fetching the content from the CDN, and increasing the QoE of the users.

### 4.2. Results

We evaluated our proposed framework for three parameters. First, we calculated the traffic flow from the CDN, i.e., transit link load for both proposed VNF based multicast and unicast transmission. Second, the traffic load on RAN is calculated, i.e., bandwidth usage. Third, we examined the usage of the total number of Resource Blocks (RBs) on the last leg of the network. Fourth, we calculated the achieved QoE of each user in both unicast and multicast transmissions.

#### 4.2.1. Transit Link Traffic

**Figure 3:**
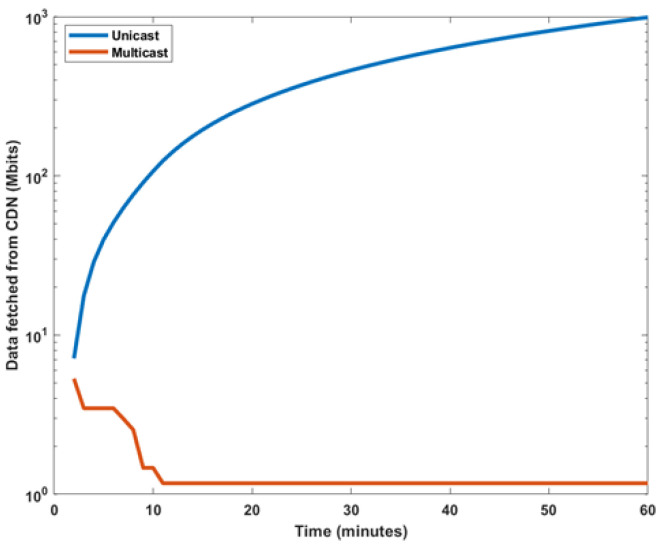
Data fetched from CDN.

Figure 3 depicts the comparison between the unicast and multicast VNF-AS modes in context to the burden on the transit link. The transit link sustains the traffic load from the CDN and towards the RAN. The figure shows that the aggregated data for all the students participating in an online session fetched from the CDN in the case of unicast mode are 6,330,300 megabits—whereas the data fetched from the CDN for the same number of students in the case of multicast transmission are 721,300 megabits. This shows a huge amount of savings for MNO in terms of cost as well as the lowered burden on the transit link for VNF-AS multicast.

#### 4.2.2. Backhaul Traffic

**Figure 4:**
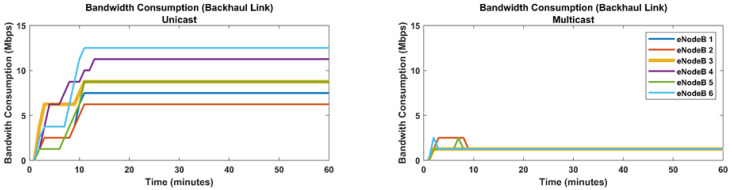
Bandwidth consumption at backhaul link unicast vs. multicast.

For the load within the core network of the MNO, we calculated the bandwidth usage at the backhaul link. Figure 4 shows massive bandwidth savings when the VNF-AS multicast transmission is exploited. The bandwidth usage at the backhaul link in the case of unicast and VNF-AS multicast modes are 2982.5 megabits/second and 460 megabits/second, respectively. Moreover, it is notable in Figure 4 that the bandwidth usage is highly dependable at the number of simultaneously active users in unicast mode. A greater number of users means higher bandwidth usage. However, in the case of multicast transmission, the number of users on a multicast channel does not affect or increase the bandwidth usage.

#### 4.2.3. QoE

**Figure 5:**
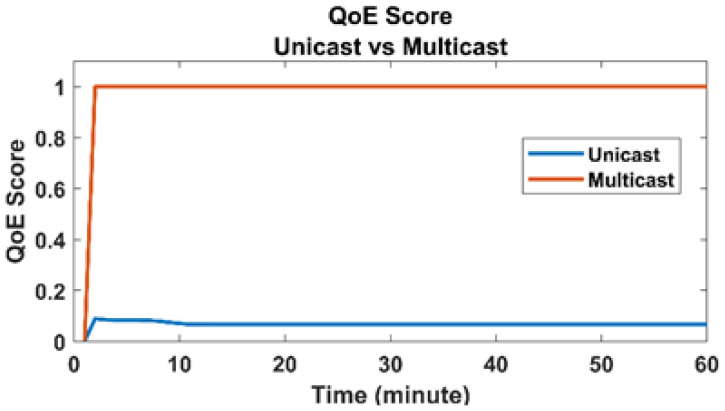
QoE score (multicast vs. unicast).

A resource block is a scarce resource and the basic data unit in LTE and 5G cellular networks. In Orthogonal Frequency Division Multiple Access (OFDMA), an eNB sends the data to a User Equipment (UE) encapsulated within multiple frames. An LTE frame is composed of various symbols or RBs. The number of RBs within a frame depends on the Channel Quality Indicator (CQI) and the Modulation and Coding Scheme (MCS) of the UE. The better the CQI/MCS, the lower the number of RBs that are required to transmit a block of data increasing the network efficiency at the access layer. If the CQI of an UE is bad, the eNB needs to assign a higher number of RBs to the UE to maintain a favorable QoE for the users. The usage of RBs and the achieved QoE are highly dependable on each other. Therefore, during our simulations, we conducted extensive experiments to specifically calculate the number of RBs assigned to a user while maintaining the optimum QoE. Figure 5 shows that, when the unicast mode is used, the collective QoE is 0.597143. On the other hand, QoE increases to a perfect 1 when the multicast transmission is exploited. These numbers show that the collective QoE of the users can be increased in the VNF based multicast mode.

#### 4.2.4. Resource Block at RAN

**Figure 6:**
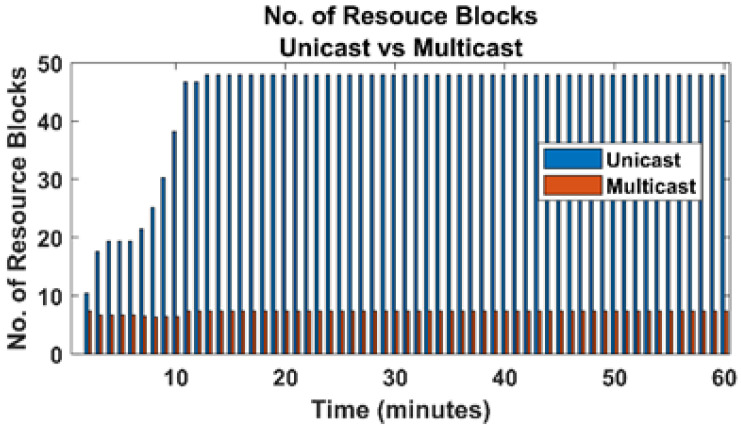
Resource blocks used (multicast vs. unicast).

As mentioned earlier, the RBs used by all the participating UEs and the QoE achieved by all those users are highly interlinked with each other. To maintain a threshold of QoE, an eNB may need to assign a greater number of RBs to a specific user with a bad CQI. In the case of unicast, each user is tuned to a dedicated physical channel. Therefore, each UE needs a specific amount of RBs on its channel. However, in the case of multicast transmission, multiple users are tuned on a shared multicast channel, making it possible for the RBs assigned to the channel to be used by all the UEs simultaneously. Figure 6 shows that, when the unicast mode is used, then the total number of RBs used by all the users is 25,730. However, the number of RBs reduces to 4360 in VNF based multicast mode. Therefore, the burden on the RAN link can also be reduced significantly in VNF based multicast mode. The results also show that predictive multicast can further increase the efficiency of VNF-AS design.

## 5. Research Challenges and Future Work

The applications of ML in wireless edge networks are transpiring consistently. Live video stream for distance learning calls for innovative and intelligent solutions for network resource management. However, the EI proposals are in the early stages with initial findings requiring further work. There are many challenges towards the proposed implementation of EI in multicast distance learning. These are listed as follows:

- **Data Availability:** Foremost, the data availability for the application of ML techniques is currently not sufficient. Very few datasets exist that reveal information regarding user sessions and preferences where the application of supervised learning is desired to predict content popularity for a region covered by an edge network [13].
- **Mobility prediction:** User mobility prediction is essential for the user-BS association and consequent edge caching decisions. Google and Apple have made mobility datasets available for pandemic studies [1]. However, the mobility datasets need to be explored for EI-based multicast solutions.
- **Community detection:** Predictive multicast requires detection of user communities requesting live and non-live videos at the MNO core. Influential users can be marked to cache live video stream for future D2D propagation to offline users. As session data are encrypted, service-less multicast solutions require the application of pre-trained ML models to transfer knowledge from relevant domains such as online social networks [33].
- **Privacy protection:** Proactive edge caching necessitates content popularity, user mobility, and content access pattern prediction to achieve high cache hit ratios. The privacy requirements of end-user necessitate that the user location and preferences data are not collected and processed in a central repository. Therefore, the application of distributed federated learning models is imperative for EI. Federated learning divides the task of machine learning on each participant node (student) avoiding the collection of data in a central repository [12].
- **Dynamics of wireless network:** As the distance learning sessions within an edge network can be numerous with dynamic wireless channel characteristics, edge cache replacement decisions demand the application of reinforcement learning [13].

In summary, the application of EI for predicting the dynamics of user and edge network is indispensable and an intricate problem with several challenges requiring further investigation.

## 6. Conclusions

This article presented a viable three-dimensional solution to augment eMBMS architecture to carry out service-less multicast in a 5G network for distance learning. We presented a network architecture that enables on-the-fly multicast transmission by using VNF-AS that can reduce the traffic burden on different levels of the 5G cellular technology without changing the major building blocks of existing standard eMBMS architecture. We conducted experiments on a real-world edge network dataset for a 720p video streaming to demonstrate the feasibility and efficacy of the proposed architecture in terms of bandwidth consumption, QoE, and resource utilization. Moreover, we debated the feasibility of EI-enabled predictive multicast and edge caching to further alleviate the burden on 5G networks due to increasing distance learning activities. Proactive edge caching can facilitate users with limited network connectivity to watch the video stream with D2D transmission. We aim to investigate user-session information at the MNO core to cluster unicast sessions for predictive multicast and edge caching.

## Figures and Tables

**Table 1:** Comparison of existing works.

Ref.	Objective	5G Architecture Changes	ML
[21]	Two layer Non-Orthogonal Multiplexing (NOM) to deliver multicast	Yes	No
[22]	Service-based method for broadcast/multicast	Yes	No
[23]	offloads video content from cellular network to dense D2D 5G networks considering the physical and social attributes	No	No
[4]	VNF-based scheme to enable multicast	No	No
[15]	CNN for popularity prediction and edge caching	NA	Yes
[25]	SVM for popularity prediction and edge caching	NA	Yes
This article	three-dimensional solution for EI enabled multicast and caching	No	Yes

## Data Availability

Not applicable.
